# Physician Knowledge and Attitudes Toward the Adoption of Peritoneal Dialysis in the Treatment of Patients With End-Stage Kidney Disease

**DOI:** 10.7759/cureus.32708

**Published:** 2022-12-19

**Authors:** Hussain S Lalani, Anisha Ganguly, Larry S Brown, Jillian Smartt, David H Johnson, Kavita P Bhavan, Ramesh Saxena

**Affiliations:** 1 Division of Pharmacoepidemiology and Pharmacoeconomics, Department of Internal Medicine, Brigham and Women's Hospital, Boston, USA; 2 Center of Innovation and Value, Parkland Health, Dallas, USA; 3 Department of General Internal Medicine, University of Texas Southwestern Medical Center, Dallas, USA; 4 Department of Biostatistics, Parkland Health, Dallas, USA; 5 Department of Internal Medicine, University of Texas Southwestern Medical Center, Dallas, USA; 6 Division of Nephrology, Department of Internal Medicine, University of Texas Southwestern Medical Center, Dallas, USA

**Keywords:** hemodialysis, peritoneal dialysis, general nephrology, internal medicine division, physician perception, knowledge attitude, survey, peritoneal dialysis (pd)

## Abstract

Introduction

Hemodialysis (HD) is a significant contributor to Medicare spending. Peritoneal dialysis (PD) is a lower-cost dialysis modality with non-inferior clinical outcomes. Recent initiatives at the federal level have emphasized shifting dialysis from in-center to home modalities, namely, PD. Such policy has been slow to impact the distribution of HD and PD due to multiple barriers, including at the provider level. Previous research has characterized the role of patient knowledge gaps and preferences in the under-utilization of PD. We sought to understand physician knowledge and attitudes toward PD to elucidate provider-level barriers to PD adoption.

Methods

We conducted a 10-question survey assessing physician comfort level, perceived knowledge, and objective knowledge of HD and PD that was distributed among the internal medicine faculty at the University of Texas Southwestern Medical Center, Dallas, TX. The survey respondents included nephrologists and non-nephrologists. Demographic information of respondents was collected. Survey responses were summarized and stratified by medical specialty. All statistical tests used 0.05 as the statistical significance level.

Results

Among 391 survey recipients, there were 83 respondents (21.2%). The mean age of respondents was 43 and 54% were women. With regard to specialty, 88% of respondents were non-nephrologists and 12% were nephrologists. All respondents reported an increased level of comfort and experience caring for patients receiving HD compared to PD. Regardless of specialty, respondents had a high incorrect response rate with regard to contraindications to PD. While nephrologists reported high perceived knowledge regarding PD, objective assessments revealed knowledge gaps with regard to PD candidacy. Non-nephrologists reported lower perceived knowledge but scored better on objective knowledge assessments regarding medical contraindications to PD. Both specialty groups held misconceptions regarding psychosocial barriers to PD.

Discussion

This physician survey demonstrated overall decreased confidence in knowledge and experience in the care of patients receiving PD compared to HD. Knowledge assessments revealed discordance between perceived knowledge and objective knowledge with regard to contraindications to PD. These findings highlight ongoing misconceptions across medical specialties regarding the applicability of PD. These findings demonstrate the need for increased training on PD candidacy among nephrologists and non-nephrologists alike. These findings demonstrate the need for education and advocacy around PD for providers to effectively meet federal priorities advocating for shifting dialysis to the home.

Conclusion

This study demonstrates the impact of physician knowledge and attitudes toward PD in the under-utilization of PD as a dialysis modality. These findings demonstrate a need for increased provider education around PD candidacy and the benefits of shifting dialysis care to the home. Novel models of dissemination are needed to increase the adoption of PD and meet federal policy goals of shifting dialysis care to home-based modalities.

## Introduction

More than 800,000 patients in the United States have been diagnosed with end-stage kidney disease (ESKD) [1]. While comprising less than 1% of Medicare beneficiaries, ESKD patients account for 7.2% of total Medicare expenditures, an estimated $37 billion annually [1]. In an effort to reduce costs and improve clinical outcomes for ESKD patients, the United States Department of Health and Human Services (DHHS) launched the Advancing American Kidney Health Initiative [2]. This effort established three federal priorities: (1) reducing the risk of developing ESKD, (2) improving access and quality of dialysis by shifting care to home, and (3) increasing access to kidney transplants (KTs). In response, the Center for Medicare and Medicaid Innovation (CMMI) proposed the End Stage Renal Disease Treatment Choices (ETC) model, a new payment model with incentives to encourage greater adoption of home dialysis and KT [3].

Peritoneal dialysis (PD) is the predominant home dialysis modality, comprising more than 80% of dialysis modalities utilized in the home [1]. Compared to in-center hemodialysis (ICHD), PD has demonstrated a lower adjusted mortality rate (135 compared to 159 per 1,000 patient-years), lower 12-month hospitalization rate after dialysis initiation, and higher median survival (55 months compared to 48 months) [1]. With regard to transplant outcomes, PD is associated with a lower risk of delayed graft function after KT [4,5]. Perhaps most importantly, PD has been consistently associated with superior patient-centered outcomes, including productivity and quality of life measures, compared to ICHD [6-8]. Despite many benefits, utilization of PD in the USA remains disappointingly low. Given that annual Medicare spending per patient is much lower for PD compared to ICHD ($81,091 compared to $94,608), underutilization of PD contributes to the Medicare cost burden of caring for patients with ESKD [1].

Given these well-established benefits, why are more patients not receiving PD? The answer is multifactorial and requires a closer look at historical policies and practices that influence physician and patient preferences in dialysis modality choice architecture [6]. Knowledge gaps around the candidacy and benefits of PD among both patients and physicians who counsel ESKD patients about dialysis contribute to the underutilization of PD [9,10], along with the highly corporatized hemodialysis (HD) industry [11]. While several studies have demonstrated decreased knowledge and awareness of dialysis modality choices among patients [12,13], the role of physician knowledge gaps and experiences is less understood. In this study, we conducted a survey of internal medicine faculty, including both non-nephrologists and nephrologists, to explore physician knowledge, awareness, and attitudes toward PD.

## Materials and methods

We developed a 10-question survey (Table 1) to assess the knowledge and attitudes around peritoneal dialysis and hemodialysis among internal medicine faculty at the University of Texas Southwestern Medical Center, Dallas, TX. Both non-nephrologists and nephrologists were included in the survey pool to compare dialysis knowledge and attitudes across disciplines, recognizing that non-nephrologists deliver a significant portion of care for dialysis patients in the primary care and inpatient settings and may influence patient dialysis choice through these encounters. The survey was created *de novo* by our study team, and correct answers for objective knowledge assessments were defined by our study team content expert in peritoneal dialysis. The survey was reviewed for validity and readability by a doctoral expert in evaluations and survey design. It included a combination of multiple-answer, multiple-choice questions, true/false, and fill-in-the-blank questions and was sent via SurveyMonkey (Momentive Inc., San Mateo, CA) to all faculty in the department. A reminder was sent to those who had not completed the survey after two weeks. No incentives for survey completion were offered.

**Table 1:** Survey on dialysis attitudes and knowledge

1. Please indicate the extent to which you agree with the following items in regard to hemodialysis.					
	1 = Strongly disagree	2 = Somewhat disagree	3 = Neutral	4 = Somewhat agree	5 = Strongly agree
I am very knowledgeable about hemodialysis					
I have been trained to perform hemodialysis					
I am very comfortable caring for patients receiving hemodialysis					
2. Please indicate the extent to which you agree with the following items in regard to peritoneal dialysis.					
	1 = Strongly disagree	2 = Somewhat disagree	3 = Neutral	4 = Somewhat agree	5 = Strongly agree
I am very knowledgeable about hemodialysis					
I have been trained to perform hemodialysis					
I am very comfortable caring for patients receiving hemodialysis					
3. Which of the following is/are absolute medical contraindications for peritoneal dialysis?					
	History of previous abdominal surgery or bowel perforation	Lack of peritoneum	Significant cognitive impairment	Cirrhosis	Heart failure
4. Which of the following is/are absolute psychosocial contraindications for peritoneal dialysis?					
	Homelessness	Having pets at home	Poor health literacy	Inability to maintain personal hygiene	Mental illness
5. In the last year, approximately how many times have you cared for dialysis patients? (Scale from 0 to 100 patient encounters)					
6. Please indicate your specialty.					
7. What is your gender?					
8. Please indicate which of the following roles best aligns with your position:					
	Physician	Physician educator	Physician researcher	Center director	Other
9. Approximately how many years have you been in practice?					
10. What is your age in years?					

Briefly, the first two questions focused on the perceived knowledge, training, and level of comfort regarding HD and PD using a Likert scale from strongly disagree to strongly agree. Question 3 evaluated the objective knowledge of the processes associated with HD and PD. Question 4 evaluated the objective knowledge of the outcomes closely associated with the two dialysis modalities. Questions 5 and 6 tested the absolute medical and psychosocial contraindications for PD. Question 7 had the respondent approximate how many times in the last year they cared for a type of dialysis patient. The remaining questions queried demographic characteristics, including specialty, role, gender, age, and years of practice. The survey responses were summarized with frequencies, percentages, means, and standard deviations and were stratified by medical specialty. Categorical questions were compared with a chi-square test and continuous questions were compared with a t-test. All statistical tests used 0.05 as the statistical significance level. The software used for the summarization and statistical analysis was SPSS (version 25, IBM Corp., Armonk, NY).

## Results

**Figure 1:**
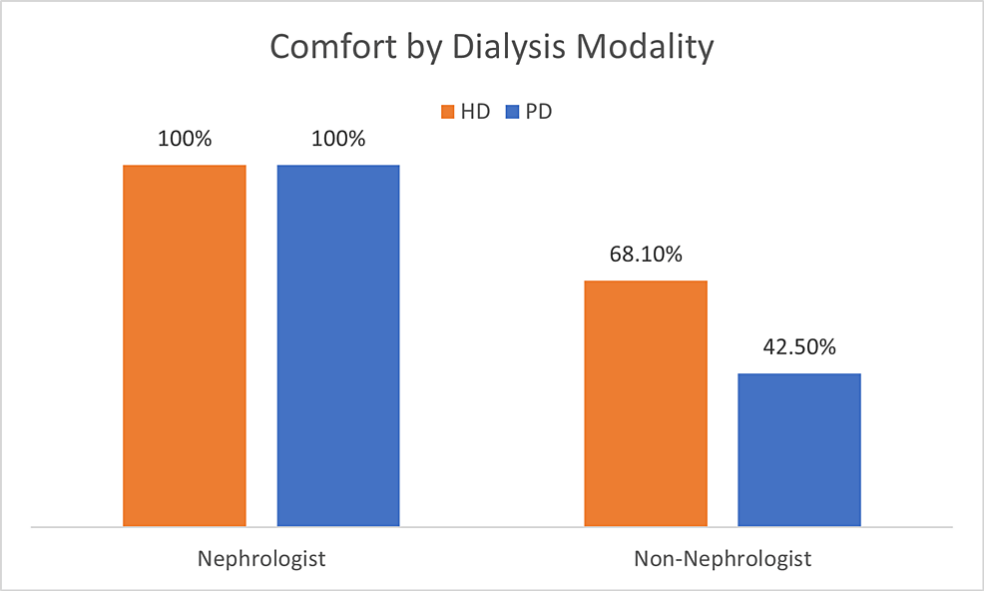
Comfort by dialysis modality among nephrologists and non-nephrologists HD: hemodialysis; PD: peritoneal dialysis.

**Figure 2:**
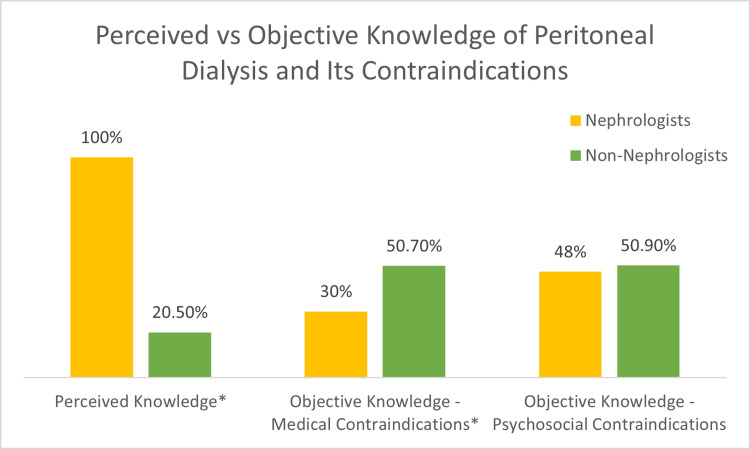
Perceived vs. objective knowledge of peritoneal dialysis and its contraindications

Of the 391 survey recipients, 83 completed the survey in its entirety (21.2%), with a response rate of 50% among nephrologists (10 responses among 20 recipients) and 19.7% among non-nephrologists (73 responses among 371 recipients). Of the 83 survey respondents, the mean age was 43 years, 54% were women, and the mean years of practice was 12.6 (Table 2). There were 10 (12%) nephrologist respondents, and the remaining 73 (88%) were from other internal medicine disciplines. Both nephrologists and non-nephrologists reported increased perceived knowledge and comfort with caring for patients on HD compared to patients on PD (Figure 1). Both groups of respondents reported an increased frequency of HD among dialysis encounters, more than three times the number of PD encounters. The majority of faculty, regardless of specialty, held misconceptions about the medical and psychosocial contraindications of PD (Figure 2). While all nephrologists felt very comfortable (100%) and knowledgeable (100%) caring for PD patients, nephrologists scored 30% correct regarding medical contraindications and 48% regarding psychosocial contraindications to PD. Interestingly, non-nephrologists scored higher regarding medical contraindications, despite lower comfort and perceived knowledge (42.5% and 20.5%) of PD.

**Table 2:** Basic demographics, knowledge, and comfort with dialysis among internal medicine faculty

	Nephrologists (N = 10)	Non-nephrologists (N = 73)	P-value
Age, mean (SD)	43 (11)	43 (12)	0.98
Years in clinical practice, mean (SD)	10 (11)	13 (12)	0.48
% female	20%	59%	***
# reported dialysis encounters in 2019, mean (SD)			
HD	98 (5)	48 (33)	<0.01
PD	34 (23)	9 (10)	<0.01
Reported comfort with patient care			
HD	100%	68.1%	0.06
PD	100%	42.5%	<0.01
Perceived knowledge			
HD	100%	37.0%	<0.01
PD	100%	20.5%	<0.01
Knowledge about absolute medical contraindications to PD (% correct answers)	30.0%	50.7%	<0.01
Abdominal surgery or bowel perforation (false)	0%	42.5%	<0.01
Lack of peritoneum (true)	100%	84.9%	0.34
Significant cognitive impairment (false)	50%	68.5%	0.29
Cirrhosis (false)	0%	45.2%	<0.01
Heart failure (false)	0%	12.3%	0.59
Knowledge about absolute psychosocial contraindications to PD (% correct answers)	48.0%	50.9%	0.73
Homelessness (true)	100%	90.3%	0.59
Pets in the home (false)	0%	5.5%	1.00
Poor health literacy (false)	40%	49.3%	0.74
Inability to maintain personal hygiene (false)	80%	76.7%	1.00
Mental illness (false)	20%	32.9%	0.49

## Discussion

In this physician survey, respondents reported decreased confidence in the knowledge of PD compared to hemodialysis and were found to have discordant measures of comfort and perceived knowledge of PD compared to objective measures of knowledge. As expected, non-nephrologists had lower comfort and perceived knowledge with regard to PD as compared to nephrologists. However, objective assessment regarding candidacy for PD revealed a potential knowledge gap among nephrologists and non-nephrologists alike.

These findings highlight an important trend in physician awareness and receptiveness toward PD in dialysis modality selection. The observed discordance between self-reported comfort and objective knowledge assessment presents an opportunity to improve understanding of advances in PD among nephrologists. Nephrologists were found to overperceive the medical contraindications of PD, even more than non-nephrologists. This may indicate disproportionate hesitancy among nephrologists toward PD, potentially related to bias toward hemodialysis, increased concern for adverse outcomes from PD, such as peritonitis, or overemphasis of contraindications toward PD in nephrology training. These findings must be interpreted in the context of their limitations, namely, the small sample size in a single-center study, including a small sample of nephrologists within the internal medicine department surveyed. Larger multi-center studies of physicians and healthcare providers are needed to contextualize our findings and better understand a frequently overlooked barrier to the adoption of PD.

Our survey underscores the importance of basic education regarding PD candidacy and the subversion of misconceptions regarding PD among clinicians, both nephrologists and non-nephrologists alike, to build a stronger culture of acceptance regarding PD across clinical settings. Physician awareness and education around the broad applicability of PD would serve to equilibrate conversations around dialysis choice and support the national commitment to shift dialysis care to the home per the Advancing American Kidney Health Initiative [2]. The recent CMMI ETC payment model initiative provides an opportunity to promote PD as a preferred treatment modality via payments to dialysis providers. Previously in 2011, the Center for Medicare and Medicaid Services (CMS) instituted the ESKD Prospective Payment System (PPS) to promote higher use of home dialysis via a bundled treatment payment including training for self-dialysis modalities [14]. Although the number of patients using PD doubled from 2008 to 2019, the overall effect size of the change was modest in modifying the national usage of dialysis modalities. For instance, in 2019, only 11% of incident ESKD patients started PD compared to 85% initiating ICHD [1]. The knowledge gap revealed by our limited survey suggests that financial incentives alone may not generate the intended impact.

In addition to physician education, there are numerous other barriers to PD adoption, such as a lack of patient awareness and education about the benefits of PD, limited hospital infrastructure to support PD programs, inconsistent availability of operators who can urgently place PD catheters, and lack of sufficient training and provision to provide PD among skilled nursing facilities and rehabilitation centers, which hinder higher utilization of PD over HD [6,10,13,15]. Each of these barriers requires careful analysis and strategies to progress toward the dissemination of PD and shifting dialysis care to the home.

One strategy may be to fund the development of regional centers of excellence for PD to train physicians, allied health professionals, and staff on what is required to deliver high-quality PD for patients. Such centers can serve as a resource to staff in surrounding areas and provide guidance regarding challenging or unique circumstances. This type of regional support network can reduce the barriers to education, thereby making it easier for physicians to embrace the management of PD patients. Interestingly, when given the opportunity, our diverse population of mostly low-income, minority patients has chosen PD nearly 40% of the time [6]. Our PD program at the University of Texas Southwestern in partnership with Davita has approximately 140 patients, comprising nearly 35% of our center’s total dialysis population compared to 11% of all prevalent dialysis patients being treated with PD nationwide [1,6].

## Conclusions

Misconceptions and differing attitudes about PD among physicians are important barriers to higher utilization of PD in the United States. It is encouraging that the federal government has recognized the tremendous burden of ESKD on the national health system and has launched an initiative to address this public health issue. The ETC model provides a compelling opportunity to put in place policies that will improve the utilization of PD to improve ESKD outcomes and reduce the financial burden on the nation’s health system. Our findings suggest that incorporating resources for provider education into such initiatives would support the national effort to increase PD utilization.
